# Physical climate risk: Stock price reactions to the historically most extreme European and United States heat waves since 1979

**DOI:** 10.1371/journal.pone.0318166

**Published:** 2025-01-24

**Authors:** Mario Schuster, Julian Krüger, Rainer Lueg

**Affiliations:** 1 Institute of Management, Accounting and Finance, Leuphana University Lüneburg, Lüneburg, Lower Saxony, Germany; 2 Department of Ocean Circulation and Climate Dynamics, GEOMAR Helmholtz Centre for Ocean Research, Kiel, Schleswig-Holstein, Germany; 3 Department of Climate Variability, Max-Planck-Institute for Meteorology, Hamburg, Germany; 4 Department of Business and Economics, University of Southern Denmark, Kolding, Syddanmark, Denmark; South China Normal University School of Economics and Management, CHINA

## Abstract

Climate change has heightened the need to understand physical climate risks, such as the increasing frequency and severity of heat waves, for informed financial decision-making. This study investigates the financial implications of extreme heat waves on stock returns in Europe and the United States. Accordingly, the study combines meteorological and stock market data by integrating methodologies from both climate science and finance. The authors use meteorological data to ascertain the five strongest heat waves since 1979 in Europe and the United States, respectively, and event study analyses to capture their effects on stock prices across firms with varying levels of environmental performance. The findings reveal a marked increase in the frequency of heat waves in the 21^st^ century, reflecting global warming trends, and that European heat waves generally have a higher intensity and longer duration than those in the United States. This study provides evidence that extreme heat waves reduce stock values in both regions, with portfolio declines of up to 3.1%. However, there are marked transnational differences in investor reactions. Stocks listed in the United States appear more affected by the most recent heat waves compared to those further in the past, whereas the effect on European stock prices is more closely tied to event intensity and duration. For the United States sample only, the analysis reveals a mitigating effect of high corporate environmental performance against heat risk. This study introduces an innovative interdisciplinary methodology, merging meteorological precision with financial analytics to provide deeper insights into climate-related risks.

## Introduction

2023 was the warmest year on record in the United States (U.S.) and the second warmest on record in Europe [1]. Climate change, driven by anthropogenic greenhouse gas emissions, is evidenced by a consistent upward trend in global mean temperatures [2]. This shift towards a warmer climate is associated with a higher occurrence of extreme weather events and an increased probability of future record-breaking heat waves [3–5]. The sixth Intergovernmental Panel on Climate Change (IPCC) report recently confirmed the high likelihood of human influence since the 1950s being the cause of increases in the frequency and the magnitude of heat waves [6]. Previous heat waves have caused physical and mental health risks [7–9], excess mortality [10, 11], and done devastating damage to natural ecosystems [12]. Heat event occurrences also result in an increase in the risk of loss of livelihoods as well as in food- and water-borne diseases [6].

Financial markets research has only recently begun to account for such climate-induced risks. Bansal et al. [13] show that rising temperature is a source of long-term economic risk and is reflected in stock prices. Accordingly, the increasing frequency and severity of extreme weather no longer passes unnoticed by the stock market [14] and investors’ interest in understanding climate risks to firm values has grown [15–17]. However, recent studies have found that investors tend to underestimate physical climate risks [18, 19]. These are risks that arise directly from the effects of changes in the climate on economic activity [20]. Moreover, the effect of extreme weather events, which fall into the category of physical climate risks, on firm values remains underexplored in the literature [21]. Venturini [22] highlights gaps in climate finance research, particularly the lack of interdisciplinary approaches or integration of meteorological methods for studying extreme weather events such as heat waves. Previous event studies in climate finance have largely overlooked primary temperature data and meteorological methodologies in assessing the impact of extreme temperatures on stock prices [23–25]. Furthermore, these studies classify portfolios by industry affiliation or location rather than by firms’ individual environmental performance, focus exclusively on the U.S., and predominantly report insignificant stock price reactions. To address the research gaps concerning stock price reactions to physical climate risks arising from meteorologically defined heat waves, portfolio clustering by corporate environmental performance, and a comparative analysis of European and U.S. markets, the authors pose the following research question: *How do stock markets react to major European and U*.*S*. *heat waves*, *taking into account corporate environmental performance*? In addressing this question, the study aims to determine whether these heat waves heighten investors’ awareness of global warming in the respective regions.

Using meteorological data, this study identifies the five most severe heat waves for Europe and the U.S., respectively, since 1979, totaling ten heat waves overall. Employing event study methodology, the study then examines how the European and U.S. stock markets reacted to these. Based on the constituents of the Stoxx Europe 600 and S&P 500, the study analyzes the significance of abnormal returns (ARs) using six event windows (EWs). The stock price effects are assessed for portfolios built upon environmental performance grading by the London Stock Exchange Group (LSEG).

The results reveal a rise in the frequency of heat waves, that heat waves in the U.S. are of lower intensity and shorter duration than those in Europe, and that both stock markets reacted negatively to the major heat waves. In Europe, the intensity and duration of heat waves drive the stock price reactions, while in the U.S., however, it is the more recent heat waves that have tended to have had a greater impact on stock prices, regardless of their intensity and duration. As expected, in the U.S. stock market, firms with high environmental performance exhibit the least selling pressure, while those with poor performance experience the most. Surprisingly, the opposite pattern is observed for Europe.

This study makes a significant contribution to research as the first to integrate an analysis of major historical heat waves with an examination of stock price reactions. By combining methodologies from meteorology and finance, it introduces a novel research approach, fostering opportunities for interdisciplinary analysis. The results of this study open new ways for investors, firm managers, and policymakers to understand the interplay between extreme weather events, environmental awareness, and investment decisions. In particular, future research could extend this interdisciplinary approach to samples from developing countries that are especially vulnerable to heat risks, as well as to the analysis of heat-sensitive sectors.

The remainder of this paper is organized as follows: The second section provides a literature review and accordingly derives the hypotheses. The third section describes the interdisciplinary approach based on the methodologies from meteorology and finance. The fourth section presents and interprets the findings of the meteorological and event study analyses. The fifth section discusses the study’s findings, comparing the results with previous research, outlining their implications for practice and contributions to research, acknowledging the study’s limitations, and suggesting avenues for future research. Lastly, the sixth section presents the conclusions.

## Literature review and hypothesis development

2023 was the world’s warmest year on record [1]. The latest IPCC report strongly indicates that human activity has significantly contributed to the rise in both the frequency and severity of heat waves [6]. Nevertheless, a research gap remains in exploring heat waves in Europe and the U.S., particularly in comparing these regions and assessing whether models about the link between global warming and heat wave occurrences hold true when analyzing historical patterns. Moving from the “current” climate to a “future” climate by shifting the mean of the whole distribution towards higher temperature values not only implies a generally warmer climate, but is also inevitably connected to a higher probability of the occurrence of extremely warm temperatures and of high record-breaking temperatures [26]. Observations confirm theoretical assumptions of an increase in the frequency and intensity of heat events due to the climate change-induced rise in the temperature mean [4, 5, 27]. Further enhanced values in terms of the probability of occurrence as well as the absolute temperature are possible through an increase in the variance of the “future” climate [28]. Climate projections using different degrees of global warming provide evidence that the increase in the global mean surface temperature is associated with an increase in heat events over Europe and North America [6, 27, 28]. Accordingly, the authors propose with *Hypothesis 1a (H1a)* that more heat events have occurred in the two regions of interest (Europe and the U.S.) in recent decades, and with *Hypothesis 1b (H1b)* that the severity of the heat waves has increased for more recent occurrences:

*H1a*: *The frequency of major European and U*.*S*. *heat waves increased in the last four decades*.
*H1b*: *The magnitude of major European and U*.*S*. *heat waves increased in the last four decades*.

Heat risk is also associated with significant financial distress for firms. Graff Zivin and Neidell [29] find that higher temperatures reduce labor productivity and Dell et al. [30] that they reduce economic growth rates. IPCC modelling scenarios predict that the U.S. and Europe will likely experience adverse effects on food production due to global warming [6]. Similarly, extreme heat events have been shown to have devastating impacts on agriculture [31] and general economic output [32]. Addoum et al. [23] confirm that extreme temperature affects earnings of various industries, while Bansal et al. [13] show that rising temperature is a source of long-term economic risk and is reflected in stock prices. Consequently, the stock market is increasingly responsive to the growing frequency and intensity of extreme weather events [14].

However, the effect of extreme weather events on stock prices is understudied [21]. Most event studies on physical climate risk events and stock price effects focus predominantly on U.S. portfolios [15, 23–25, 33, 34] and there is a notable lack of European samples. Europe provides an interesting setting due to its pioneering role in environmentalism and climate protection as outlined in Teutrine et al. [35], which may indicate a high investor awareness regarding climate risks in this region. Reboredo and Ugolini [36] provide evidence that European stocks are generally more sensitive to climate risks than U.S. stocks, but Schuster et al. [37] find the opposite pattern. Thus, the research field remains inconclusive to date.

Building on the existing literature, the authors propose that the impact of heat waves on stock prices reflects the physical climate risk exposure of firms [21, 22]. Previous studies suggest that physical climate risk events can result in economic damage and that such events, accordingly, negatively influence the stock market. Bourdeau-Brien and Kryzanowski [24] reveal that extreme weather events increase stock volatility in the U.S. market and Griffin et al. [21] demonstrate that U.S. firms experience negative market responses during extreme high surface temperature events, and that these effects are intensified by extended durations of such events. Moreover, recent research provides evidence that investors’ awareness of climate change increases when physical climate risks are prominent. Alekseev et al. [38] analyzed Google trends and demonstrated that extreme heat events positively impact climate change awareness. This heightened awareness can result in stock market overreactions, as evidenced by recent findings in the context of natural disasters in the U.S. [39, 40]. The enhanced climate concerns tend to lead to negative investor responses in the stock market [21, 37]. Overall, the intensity and duration of heat events likely influence not only the extent of economic damage but also the level of public attention to heat risk, both of which, in turn, affect the degree of negative impact on stock prices. Consequently, the authors derive *Hypothesis 2a (H2a) and Hypothesis 2b (H2b)*:

*H2a*: *Major European and U*.*S*. *heat waves resulted in more negative than positive stock price reactions*.
*H2b*: *The strength of stock price reactions to heat waves depended on the intensity and duration of the heat events*.

Additionally, the enhanced sensitization to global warming is considered to be incorporated by investors in investment policies, leading to a preference for firms with high environmental responsibility. This pattern is confirmed for the U.S. stock market [40, 41]. Nevertheless, the European stock market may be even more sensitized to global warming than the U.S. market: European Union regulations aimed at combating climate change are making firms’ environmental responsibility directly financially relevant to investors [42]. Investors in the U.S. stock market tend to be more skeptical about the financial benefits of green investing [37] and have generally shown less commitment to incorporating firms’ environmental performance into their investment decisions, largely due to less stringent climate policies [43, 44].

Joireman et al. [45], Deryugina [46], and Rüttenauer [47] find that high temperatures positively influence belief in global warming. Especially in such periods, the level of firms’ environmental responsibility may influence the impact of heat risk on stock prices. From an asset-pricing perspective, low-carbon portfolios are linked to lower expected returns, driven by (sustainable) investors’ non-pecuniary preference to accept reduced returns for holding green stocks [48, 49]. However, previous research shows that green stocks outperform in the short term when the market is salient to climate risks [50–52]. Ardia et al. [53] show that when media attention concerning physical climate risk is high, green stock values increase while brown stock values decrease. Choi et al. [41] find that Google searches related to global warming increase during periods of unusually high temperatures and that carbon-intensive firms underperform compared to low-emitting firms. Moreover, Huynh and Xia [40] show that U.S. listed firms with high environmental performance experience lower selling pressure during periods with high investor awareness to natural disasters. Complementing this, Ai and Gao [54] and Barbera-Marine et al. [55] confirm that firms’ environmental performance has a mitigating effect on their exposure to climate risks. Further studies provide evidence that green stocks outperform the market after climate-related shocks [19, 41]. Accordingly, it is expected that investors also consider firms’ existing levels of environmental responsibility when making investment decisions in response to, as yet under-researched, heat risk events. Following recent findings by Schuster et al. [37], the authors derive *Hypothesis 3 (H3)*, that portfolios with superior environmental performance outperform their peer portfolios when climate risk events are salient to investors.

*H3*: *Firms with high environmental performance outperformed those with lower performance during heat waves*.

## Materials and methods

### Meteorological identification of heat waves

The large number of parameters involved in describing a heat wave (e.g., intensity, frequency, duration, spatial extent) has and continues to pose a great challenge for the scientific community in agreeing on a universally applicable heat wave metric. Consequently, various definitions exist, focusing either on a single or multiple parameters [56]. This study employs a multi-parameter-based heat wave definition, the Heat Wave Magnitude Index daily (HWMId), introduced by Russo et al. [57] and applied in studies thereafter [e.g., 58, 59]. The HWMId is based on the daily maximum temperature data (TX) from 1979 onward—when reliable reanalysis data became available as a result of satellite coverage by the ERA5 reanalysis product. The data are provided by the European Centre for Medium-Range Weather Forecasts and described in Hersbach et al. [60]. The temperature data of the ERA5 reanalysis are defined at grid points that have a uniform horizontal coverage with a resolution of 1.5° longitude x 1.5° latitude. The HWMId is defined for each grid point of the European and U.S. land masses. A heat wave is defined on a single grid point if TX exceeds a daily threshold for at least three consecutive days. The daily threshold refers to the 90^th^ percentile of the reference data set *A*_*d*_ representing the union of all TX within the 32-year long reference period from 1979 to 2010 and within a 31-day window centered on the day d:

$$A_{d}=\overset{2010}{\underset{y=1979}{\cup }}\;\overset{d+15}{\underset{i=d-15}{\cup }}T_{y,i}$$

Once a heat event is identified, the corresponding daily heat wave magnitudes *M*_*d*_ for a specific grid point are defined as follows:

$$M_{d}\left(T_{d}\right)=\left\{\right.\begin{array}{cc} \frac{T_{d}-T_{32y25p}}{T_{32y75p}-T_{32y25p}} & if\;T_{d}>T_{32y25p}\\ 0 & if\;T_{d}\leq T_{32y25p} \end{array}$$

where *T*_*d*_ refers to TX on a particular heat wave day and *T*_*32y25p*_ and *T*_*32y75p*_ represent the 25^th^ and 75^th^ percentile of the distribution of the 32 annual maximum temperatures within the reference period of 1979 to 2010. The unit of *M*_*d*_ is expressed as the magnitude relative to the interquartile range of the distribution of the 32 annual maximum temperatures. The total magnitude of all heat waves identified in a year is obtained by summing up the daily magnitudes *M*_*d*_ for all days comprising a heat wave, respectively. The HWMId refers to the particular heat wave with the highest total magnitude in a year. Hence, the HWMId measure combines duration (days comprising a heat wave) and intensity (daily heat wave magnitude *M*_*d*_). This study considers the top five heat waves in Europe and the U.S., respectively. These are identified using the grid point with the highest annual HWMId (maximum HWMId) as the primary condition. A sufficient spatial extent serves as a secondary condition (at least 10% of the grid points are required to have a HWMId >6 relative to all grid points encompassing the European or U.S. domain). Through this approach, the study intentionally excludes small-scale phenomena that are not considered to be high-impact heat waves.

### Event study approach

The authors employ the event study methodology proposed by MacKinlay [61] and utilize the corresponding Stata codes provided by Ullah et al. [62]. Accordingly, the authors follow the following steps: (1) define events, EWs, and portfolios; (2) estimate normal returns; (3) calculate ARs; and, (4) test the portfolios for statistical significance.

First, the study identifies the strongest heat wave for each year represented by the HWMId, selecting the top five in each geographical region since 1979. The day of the strongest daily heat wave magnitude *M*_*d*_ within the respective top five heat waves is defined as the heat wave peak and serves as the respective event date. A time window around the event date defined in this manner is most likely associated with the period of the strongest attention to this particular heat event [63]. The same six EWs surrounding each heat wave peak are tested: [−5, −1], [−3, +1], [−1, +1], [−1, +3], [+1, +5], and [−5, +5]. This allows for the comparison of heat waves with varying durations. The sample is constructed based on firms listed in the Stoxx Europe 600 and S&P 500, using LSEG Eikon as the data source for daily stock returns. For each event date, the constituents of both indices are clustered according to the environmental performance grades from LSEG (assessed using three categories: resource use, emissions, and environmental innovation), classified in the range of A+ to D- based on previously calculated environmental scores [64]. Following Birindelli and Chiappini [42] and Schuster et al. [37], the authors categorize grade A as a high score, B as medium, and C and D as low. As an illustrative overview of the sample distribution, for the year 2021, most European firms were assessed as high scorers, while most U.S. firms were medium scorers (Table 1).

**Table 1:** CAARs as per environmental grade portfolios.

**Panel A—EW [−5, −1]**									
**Europe**					**U.S.**				
	**N**	**CAAR**	**T-test**	**Wilcoxon**		**N**	**CAAR**	**T-test**	**Wilcoxon**
**2022**					**2023**				
High Score	285	-0.158%	0.469	0.567	High Score	163	-0.523%***	0.003	0.000
Medium Score	187	0.303%	0.364	0.147	Medium Score	208	-0.334%**	0.039	0.084
Low Score	126	1.997%***	0.000	0.000	Low Score	127	-0.296%	0.315	0.232
**2021**					**2021**				
High Score	262	-0.608%***	0.003	0.006	High Score	146	-1.248%***	0.000	0.000
Medium Score	197	-0.353%	0.131	0.532	Medium Score	171	-0.706%***	0.001	0.000
Low Score	129	-0.560%	0.115	0.183	Low Score	164	-0.716%***	0.003	0.000
**2018**					**2012**				
High Score	218	-0.231%	0.231	0.256	High Score	69	0.170%	0.709	0.643
Medium Score	168	0.509%**	0.017	0.001	Medium Score	113	0.183%	0.604	0.132
Low Score	140	1.355%***	0.000	0.000	Low Score	234	-0.189%	0.590	0.132
**2014**					**2011**				
High Score	164	0.251%	0.308	0.489	High Score	67	0.030%	0.933	0.529
Medium Score	150	-0.334%	0.126	0.035	Medium Score	102	-0.193%	0.582	0.735
Low Score	129	0.081%	0.762	0.741	Low Score	238	-1.534%***	0.000	0.000
**2010**					**2007**				
High Score	145	0.185%	0.440	0.338	High Score	10	-1.183%	0.364	0.375
Medium Score	113	0.044%	0.885	0.930	Medium Score	47	-1.521%***	0.001	0.001
Low Score	147	0.015%	0.959	0.852	Low Score	272	-0.464%*	0.059	0.038
**Panel B—EW [−3, +1]**									
**Europe**					**U.S.**				
	**N**	**CAAR**	**T-test**	**Wilcoxon**		**N**	**CAAR**	**T-test**	**Wilcoxon**
**2022**					**2023**				
High Score	285	-1.316%***	0.000	0.000	High Score	163	0.069%	0.667	0.599
Medium Score	187	-1.103%***	0.009	0.003	Medium Score	208	0.141%	0.409	0.312
Low Score	126	0.073%	0.832	0.790	Low Score	127	0.393%	0.494	0.990
**2021**					**2021**				
High Score	262	-0.883%***	0.000	0.000	High Score	146	-0.584%**	0.014	0.004
Medium Score	197	-0.213%	0.335	0.390	Medium Score	171	-0.449%**	0.023	0.002
Low Score	129	0.623%	0.125	0.436	Low Score	164	-0.661%***	0.002	0.000
**2018**					**2012**				
High Score	218	0.061%	0.774	0.643	High Score	69	-0.048%	0.925	0.395
Medium Score	168	0.025%	0.933	0.821	Medium Score	113	0.390%	0.232	0.014
Low Score	140	0.820%***	0.009	0.005	Low Score	234	-0.717%*	0.051	0.003
**2014**					**2011**				
High Score	164	0.402%	0.095	0.107	High Score	67	-0.401%	0.320	0.914
Medium Score	150	-0.021%	0.920	0.629	Medium Score	102	-0.457%	0.226	0.367
Low Score	129	-0.101%	0.712	0.252	Low Score	238	-0.585%*	0.080	0.051
**2010**					**2007**				
High Score	145	0.017%	0.936	0.947	High Score	10	-0.504%	0.673	0.492
Medium Score	113	-0.502%**	0.043	0.031	Medium Score	47	-1.458%***	0.000	0.001
Low Score	147	0.020%	0.929	0.846	Low Score	272	-0.529%**	0.047	0.070
**Panel C—EW [−1, +1]**									
**Europe**					**U.S.**				
	**N**	**CAAR**	**T-test**	**Wilcoxon**		**N**	**CAAR**	**T-test**	**Wilcoxon**
**2022**					**2023**				
High Score	285	-0.887%***	0.000	0.000	High Score	163	-0.252%**	0.044	0.020
Medium Score	187	-1.788%***	0.000	0.000	Medium Score	208	-0.213%*	0.071	0.042
Low Score	126	-1.874%***	0.000	0.000	Low Score	127	-0.020%	0.970	0.003
**2021**					**2021**				
High Score	262	-0.570%***	0.000	0.001	High Score	146	-0.888%***	0.000	0.000
Medium Score	197	-0.185%	0.343	0.775	Medium Score	171	-0.541%***	0.003	0.000
Low Score	129	0.806%**	0.016	0.017	Low Score	164	-1.217%***	0.000	0.000
**2018**					**2012**				
High Score	218	0.435%**	0.015	0.001	High Score	69	-0.568%	0.098	0.172
Medium Score	168	0.188%	0.424	0.885	Medium Score	113	0.409%**	0.029	0.030
Low Score	140	0.511%*	0.056	0.021	Low Score	234	-0.746%***	0.000	0.000
**2014**					**2011**				
High Score	164	0.144%	0.356	0.534	High Score	67	-0.465%	0.172	-0.625
Medium Score	150	-0.053%	0.716	0.387	Medium Score	102	-0.429%	0.150	0.128
Low Score	129	-0.445%**	0.011	0.007	Low Score	238	-0.551%**	0.023	0.026
**2010**					**2007**				
High Score	145	-0.209%	0.149	0.229	High Score	10	-0.241%	0.812	0.492
Medium Score	113	-0.884%***	0.000	0.000	Medium Score	47	-1.462%***	0.000	0.000
Low Score	147	-0.446%**	0.010	0.002	Low Score	272	-0.406%*	0.094	0.023
**Panel D—EW [−1, +3]**									
**Europe**					**U.S.**				
	**N**	**CAAR**	**T-test**	**Wilcoxon**		**N**	**CAAR**	**T-test**	**Wilcoxon**
**2022**					**2023**				
High Score	285	-0.651%***	0.008	0.015	High Score	163	-0.614%***	0.000	0.000
Medium Score	187	-1.199%***	0.000	0.002	Medium Score	208	-0.631%***	0.003	0.000
Low Score	126	-0.267%	0.484	0.363	Low Score	127	-0.341%	0.486	0.000
**2021**					**2021**				
High Score	262	0.115%	0.481	0.345	High Score	146	-1.680%***	0.000	0.000
Medium Score	197	0.032%	0.892	0.270	Medium Score	171	-1.484%***	0.000	0.000
Low Score	129	0.843%**	0.016	0.002	Low Score	164	-2.178%***	0.000	0.000
**2018**					**2012**				
High Score	218	-0.075	0.751	0.856	High Score	69	-0.442%	0.323	0.392
Medium Score	168	-0.028%	0.889	0.848	Medium Score	113	0.363%	0.162	0.212
Low Score	140	0.657%**	0.037	0.014	Low Score	234	-0.902%***	0.000	0.000
**2014**					**2011**				
High Score	164	-0.149%	0.574	0.418	High Score	67	-0.275%	0.524	0.948
Medium Score	150	-0.430%*	0.065	0.018	Medium Score	102	-0.093%	0.818	0.984
Low Score	129	-0.300%	0.244	0.100	Low Score	238	0.611%*	0.058	0.010
**2010**					**2007**				
High Score	145	-1.297%***	0.000	0.000	High Score	10	-0.314%	0.697	0.160
Medium Score	113	-1.814%***	0.000	0.000	Medium Score	47	-1.288%***	0.002	0.001
Low Score	147	-1.846%***	0.000	0.000	Low Score	272	-0.152%	0.553	0.241
**Panel E—EW [+1, +5]**									
**Europe**					**U.S.**				
	**N**	**CAAR**	**T-test**	**Wilcoxon**		**N**	**CAAR**	**T-test**	**Wilcoxon**
**2022**					**2023**				
High Score	285	0.040%	0.881	0.938	High Score	163	-0.922%***	0.000	0.000
Medium Score	187	0.074%	0.829	0.438	Medium Score	208	-0.973%***	0.000	0.000
Low Score	126	1.255%***	0.003	0.001	Low Score	127	-0.733%**	0.025	0.001
**2021**					**2021**				
High Score	262	0.017%	0.913	0.904	High Score	146	-1.553%***	0.000	0.000
Medium Score	197	0.221%	0.373	0.623	Medium Score	171	-1.923%***	0.000	0.000
Low Score	129	1.107%***	0.003	0.001	Low Score	164	-2.051%***	0.000	0.000
**2018**					**2012**				
High Score	218	-0.590%***	0.005	0.004	High Score	69	-0.155%	0.649	0.419
Medium Score	168	-0.526%*	0.056	0.002	Medium Score	113	-0.158%	0.594	0.074
Low Score	140	-0.467%	0.111	0.011	Low Score	234	-1.091%***	0.000	0.000
**2014**					**2011**				
High Score	164	-0.155%	0.561	0.361	High Score	67	0.095%	0.845	0.938
Medium Score	150	-0.508%	0.105	0.067	Medium Score	102	-0.213%	0.591	0.632
Low Score	129	0.215%	0.418	0.383	Low Score	238	1.936%***	0.000	0.000
**2010**					**2007**				
High Score	145	-1.492%***	0.000	0.000	High Score	10	0.087%	0.888	0.922
Medium Score	113	-1.268%***	0.000	0.000	Medium Score	47	1.044%***	0.007	0.024
Low Score	147	-1.597%***	0.000	0.000	Low Score	272	0.496%**	0.010	0.023
**Panel F—EW [−5, +5]**									
**Europe**					**U.S.**				
	**N**	**CAAR**	**T-test**	**Wilcoxon**		**N**	**CAAR**	**T-test**	**Wilcoxon**
**2022**					**2023**				
High Score	285	-0.994%***	0.004	0.011	High Score	163	-1.287%***	0.000	0.000
Medium Score	187	-0.611%	0.237	0.238	Medium Score	208	-1.291%***	0.003	0.000
Low Score	126	1.785%***	0.007	0.013	Low Score	127	-0.940%*	0.051	0.005
**2021**					**2021**				
High Score	262	-0.598%**	0.021	0.035	High Score	146	-3.065%***	0.000	0.000
Medium Score	197	-0.037%	0.921	0.746	Medium Score	171	-2.768%***	0.000	0.000
Low Score	129	1.167%**	0.042	0.024	Low Score	164	-2.937%***	0.000	0.000
**2018**					**2012**				
High Score	218	-0.639%**	0.031	0.039	High Score	69	-0.120%	0.858	0.826
Medium Score	168	0.107%	0.784	0.580	Medium Score	113	0.117%	0.794	0.557
Low Score	140	0.872%**	0.016	0.023	Low Score	234	-1.734%***	0.000	0.000
**2014**					**2011**				
High Score	164	0.123%	0.755	0.924	High Score	67	-0.335%	0.476	0.754
Medium Score	150	-0.959%**	0.015	0.007	Medium Score	102	-0.487%	0.305	0.357
Low Score	129	-0.064%	0.869	0.367	Low Score	238	-0.345%	0.389	0.672
**2010**					**2007**				
High Score	145	-1.466%***	0.000	0.000	High Score	10	-1.215%	0.411	0.375
Medium Score	113	-1.604%***	0.000	0.000	Medium Score	47	-1.380%**	0.010	0.006
Low Score	147	-1.732%***	0.000	0.000	Low Score	272	-0.374%	0.244	0.265

This table shows the event study results for the defined EWs. The stock price effects are listed according to the environmental performance grade portfolios for each region. The table depicts the CAARs on the last day of the respective EWs. The parametric two-sided t-test and the nonparametric Wilcoxon signed-rank test are performed. The p-values are listed in this table.

*, ** and *** indicate statistical significance at the 10%, 5% and 1% levels respectively.

If the significance levels differ between the two tests, the authors rely on the parametric t-test to mark these levels. Results are only marked as statistically significant if the p-values of both tests are below the threshold of the 10% significance level.

Second, the authors compute the normal returns with the market model which assumes a linear relation between the security and the market return [65]:

$$\begin{array}{l} \text{E}\left({\text{R}}_{\text{it}}\right)=\alpha_{\text{i}}+\beta_{\text{i}}{\text{R}}_{\text{mt}}+\varepsilon_{\text{it}}\\ E(\varepsilon_{it}=0),\;var(\varepsilon_{it})=\sigma_{\varepsilon_{i}}^{2}, \end{array}$$ (1)

where *E(R*_*it*_*)* represents the expected return on security *i* on day *t*; *R*_*mt*_ the return on the market portfolio; *α*_*i*_ the alpha as the mean return over the period not explained by the market; *β*_*i*_ the beta of firm *i*; and *ε*_*it*_ the zero mean disturbance term. Model coefficients are estimated from the estimation window, which ranges from -150 to -51 days preceding the event dates. Thus, the estimation period takes place before the start of the respective heat waves. Accordingly, the authors run a regression with daily stock returns for each firm *i*, *R*_*it*_, on the market return, *R*_*mt*_, which is proxied by the Stoxx Europe 600 and the S&P 500, respectively.

Third, the authors calculate the abnormal return (AR) for each firm *i*, which is defined as the residual between expected normal and actual stock return at time *t* [61]:

$${\text{AR}}_{\text{it}}={\text{R}}_{\text{it}}-\text{E}\left({\text{R}}_{\text{it}}\right)={\text{R}}_{\text{it}}-\left(\alpha_{\text{i}}+\beta_{\text{i}}{\text{R}}_{\text{mt}}\right)$$ (2)

where *AR*_*it*_ represents the AR of *i* on day *t*. Thereafter, the ARs are accumulated for each firm *i* to produce cumulative abnormal returns (CARs) with *t*_*1*_, *t*_*2*_ referring to the EW thresholds:

$${\text{CAR}}_{\text{i}}({\text{t}}_{1},\;{\text{t}}_{2})=\sum_{\text{t}={\text{t}}_{1}}^{{\text{t}}_{2}}{\text{AR}}_{\text{it}},$$ (3)

Fourth, the authors test the null hypothesis *H*_*0*_ [61]. To test the statistical significance of the CARs, a two-sided t-test is performed. The parametric test examines *H*_*0*_ by indicating that the CARs are zero:

$$\theta_{t}=\frac{CAR_{t}}{\sqrt{var\left(CAR_{t}\right)}\sqrt{n}}∼N\left(0,1\right),$$ (4)

*N* is the number of days in the respective EW. To evaluate the average effect of the heat waves on the various environmental grade portfolios, the cumulative average abnormal return (CAAR) for the three groups (A-rated, B-rated, and C- and D-rated) is computed. The CAAR is defined as the mean of the CARs [66]:

$${\text{CAAR}}_{\text{t}}=\frac{1}{\text{N}}\sum_{i=1}^{N}{\text{CAR}}_{\text{t}},$$ (5)

*N* represents the number of firms in each portfolio. The focus is on the CAARs at the end of each EW [67]. To ensure the robustness of the results through nonparametric testing, the Wilcoxon signed-rank test is applied [68].

## Results

### Meteorological analysis

**Fig 1:**
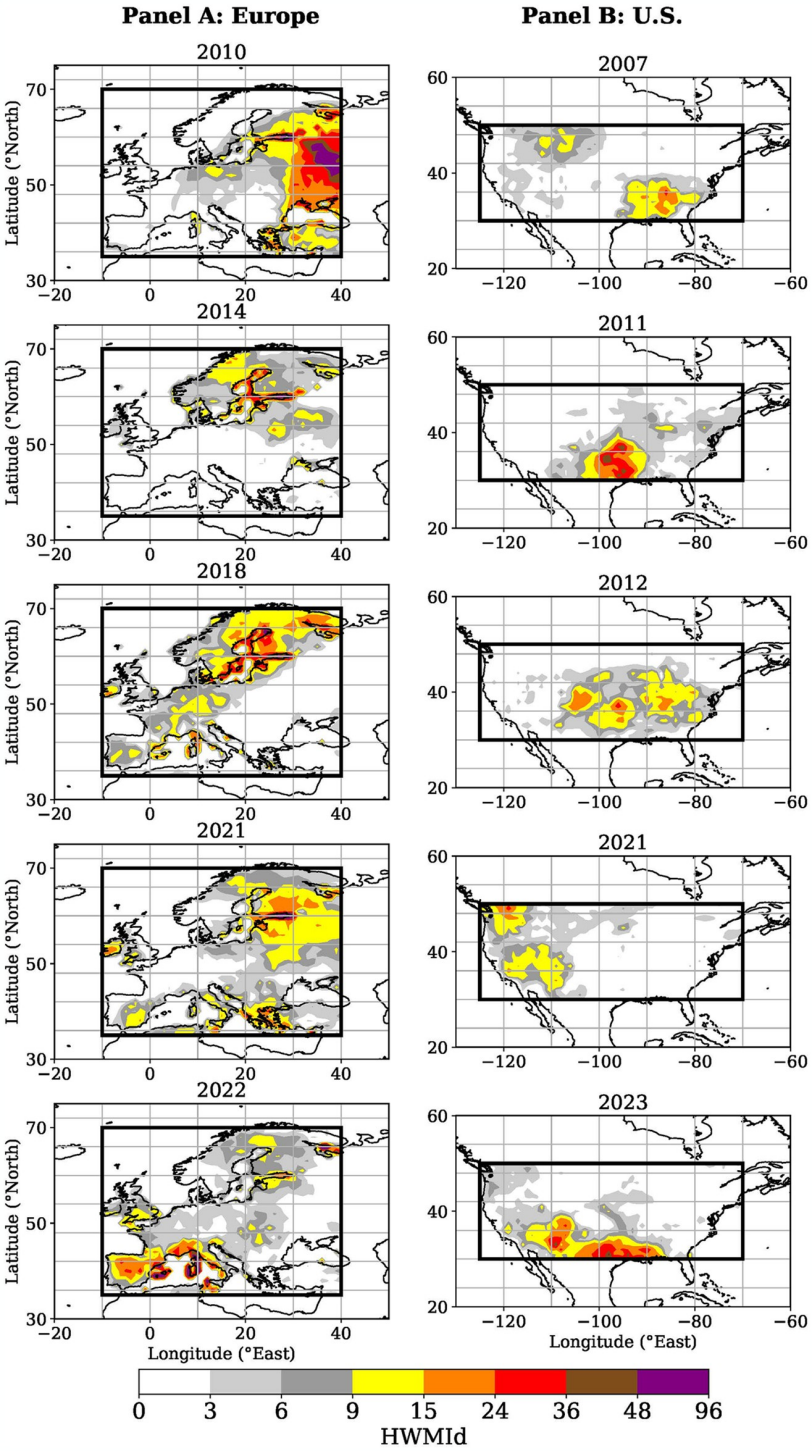
Major heat wave identification with data from 1979 to 2023. This figure shows the HWMId of the top five heat waves identified for each grid point on land within the European (10°W-40°E, 35–70°N) and the U.S. (70–125°W, 30–50°N) domains. The maps were created using the PlateCarree projection from the Cartopy Python package. Additional details for creating the figure can be found in the study’s minimal data set (see S1 File).

**Fig 2:**
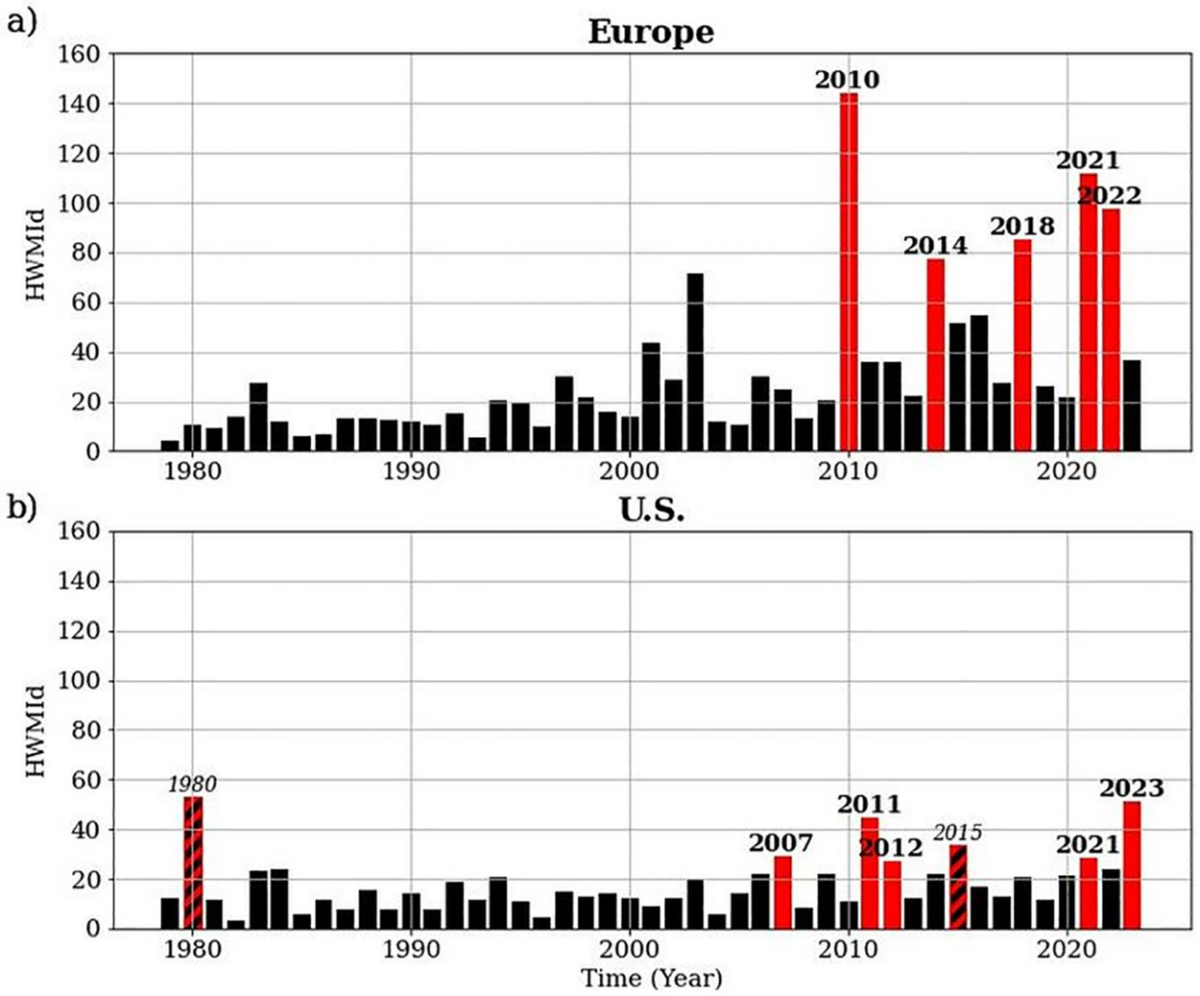
Maximum HWMId for 1979–2023 heat waves. This figure shows the maximum HWMId, i.e., the HWMId for the grid point with the highest HWMId, for each year for Europe (a) and the U.S. (b). The red bars highlight the identified top five heat waves for the respective domain. The U.S. 2015 heat wave is excluded due to insufficient spatial extent and the 1980 one due to the limited availability of stock market data.

The top five European heat waves occurred in 2010, 2014, 2018, 2021, and 2022 (Figs 1 Panel A and 2a). The 2022 heat wave is included due to its exceptionally long duration rather than its daily heat wave magnitudes (*M*_*d*_) which are relatively low compared with the other four (Table 2). The top five European heat waves all occurred in the past two decades (Fig 2a). This confirms a tendency towards a higher frequency of heat waves over the European continent [69]. Accordingly, *H1a* is accepted for Europe due to the increasing heat wave frequency resulting from climate change. *H1b*, however, must be rejected since the 2010 heat wave had a higher magnitude (HWMId value: 144.2) than the more recent top five heat events listed in Table 2.

**Table 2:** Aggregated results per heat wave.

**European heat waves**					
**Year**	**Start date**	**Duration**	**Date *M***_***d***_ **peak**	**Max. HWMId**	**Market reaction**
2010	5 July 2010	45	8 August 2010	144.2	12
2014	18 July 2014	28	28 July 2014	77.0	3
2018	12 July 2018	31	17 July 2018	84.9	10
2021	18 June 2021	32	16 July 2021	111.5	8
2022	3 July 2022	77	26 July 2022	97.4	11
**U.S. heat waves**					
**Year**	**Start date**	**Duration**	**Date *M***_***d***_ **peak**	**Max. HWMId**	**Market reaction**
2007	4 August 2007	25	16 August 2007	29.2	10
2011	16 July 2011	24	6 August 2011	44.8	5
2012	28 July 2012	8	30 July 2012	27.4	6
2021	26 June 2021	9	29 June 2021	28.4	18
2023	26 July 2023	34	27 August 2023	51.2	12

This table lists the identified top five heat waves with descriptive information for each geographical region. The table includes the following information for each of the heat waves: year; details of the grid point with the highest annual HWMId (start date, duration in days, day of peak of daily heat wave magnitudes *M*_*d*_, maximum HWMId value); and, the aggregated number of statistically significant stock market reactions across all EWs per region.

The top five heat waves identified in the U.S. occurred in 2007, 2011, 2012, 2021, and 2023 (Figs 1 Panel B and 2b). In general, U.S. heat waves are characterized by lower HWMId values than European ones (Fig 2). This phenomenon is explained by U.S. heat waves’ shorter durations (Table 2). The most severe U.S. heat wave took place in 2023 (Fig 2b and Table 2). The top five U.S. heat waves also took place in the 21^st^ century, suggesting an imprint of climate change in this domain as well (Fig 2). Hence, *H1a* is also accepted for the U.S. Considering the 2023 heat event, *H1b* can further be accepted for the U.S., as this heat wave, the most recent, has the highest magnitude of the top five heat waves (HWMId value of 51.2, see Table 2).

### Stock price effects

**Fig 3:**
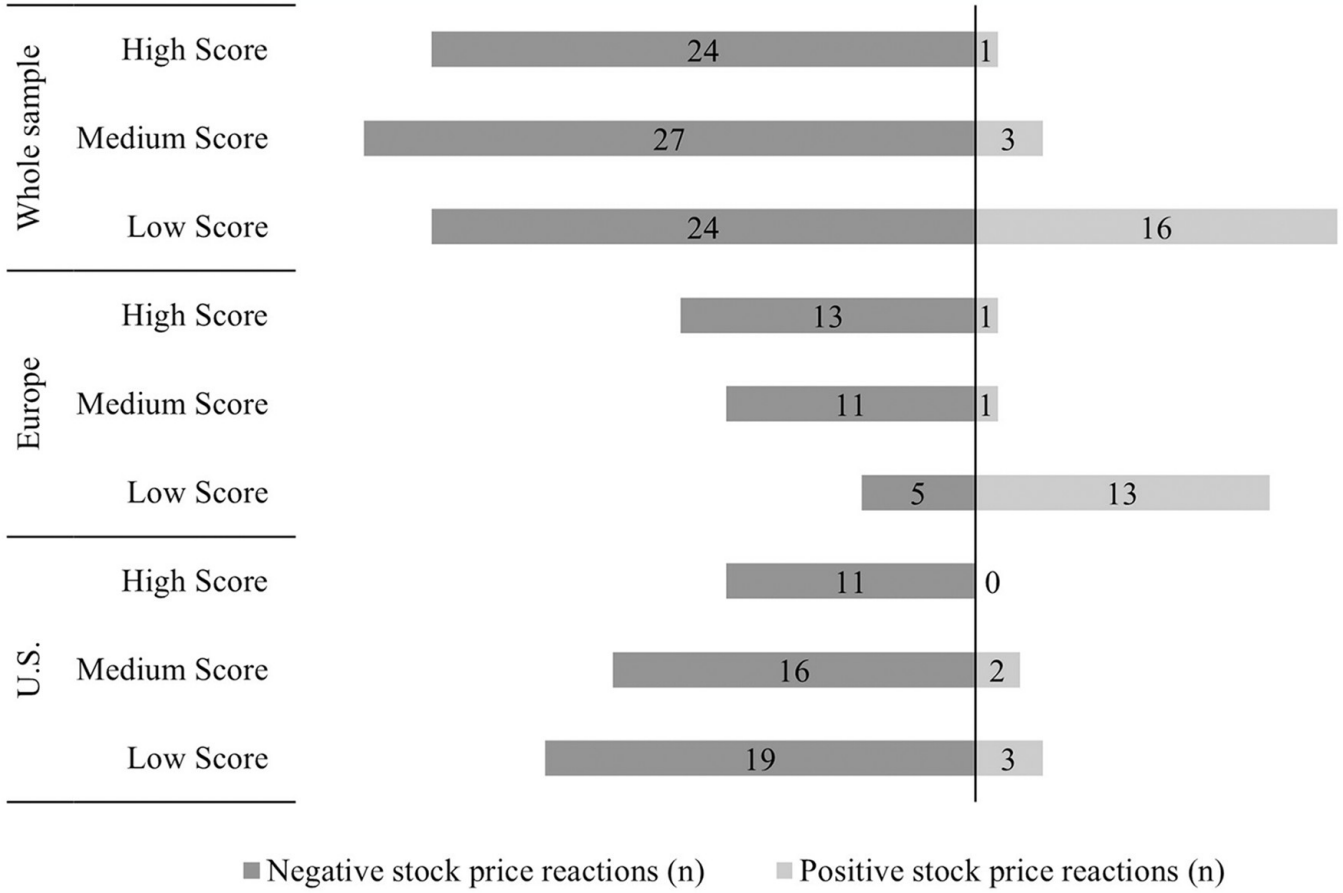
Aggregated results: Environmental grade portfolios. This figure presents an aggregated view of statistically significant stock price reactions, categorized according to environmental grade portfolios. The delineation covers all identified heat waves and EWs and is segmented into the whole sample, Europe, and the U.S. Statistically significant negative CAARs are listed as negative stock price reactions. Statistically significant positive CAARs are listed as positive stock price reactions.

The authors report statistically significant stock price reactions to each of the top five European heat waves. Encompassing all event windows, heat events, and portfolios, more negative than positive statistically significant stock price reactions are observed (Table 1 and Fig 3). Therefore, *H2a* is confirmed. The CAARs vary between 1.997% (EW [−5, −1], 2022, low-score portfolio) and -1.874% (EW [−1, +1], 2022, low-score portfolio) (Table 1). Table 2 summarizes and compares all heat waves by HWMId maximum, duration, and the number of statistically significant stock market reactions. Interestingly, as presented in Table 2, the stock market reactions depend on the severity of European heat waves: the highest number of statistically significant CAARs occurred for the 2010 (highest maximum HWMId) and the 2022 (longest duration) heat waves. Accordingly, *H2b*, which states that stock price reactions depend on the duration and intensity of heat waves, is confirmed for Europe. In contrast, the timing (year) of the heat waves does not seem to influence the effect size or frequency of statistically significant CAARs (Tables 1 and 2). Therefore, investors’ reactions to European heat waves do not appear to have increased for more recent events. Surprisingly, firms with poor environmental performance were barely penalized by investors. In fact, the most positive stock price reactions occurred for firms with low environmental performance and the most negative for firms with high environmental performance (Fig 3). Thus, overall, European firms with superior environmental performance experience stock underperformance during heat waves. This leads to a rejection of *H3*, proposing an outperformance by environmentally high-scoring firms, for Europe. The underperformance may be attributed to a dilution effect of corporate environmental performance in Europe due to the comparatively high proportion of European firms in the high-scoring portfolio. It should be noted that in 2010 and 2014 the number of firms in each environmental grade portfolio was more equally distributed. During these two European heat waves, the expected mitigation effect of high environmental performance was evident (Table 1).

Statistically significant stock price effects are identified for all U.S. heat waves (Table 1). The frequency of statistically significant CAARs is even higher than for Europe, although the maximum HWMId and the duration of the heat waves are on a lower level (Tables 1 and 2 and Fig 3). The tendency for more negative than positive statistically significant stock price reactions is even more pronounced in the U.S. than in Europe, thus also supporting *H2a* for the U.S (Table 1 and Fig 3). The CAARs range from 1.936% (EW [+1, +5], 2011, low-score portfolio) to -3.065% (EW [−5, +5], 2021, high-score portfolio) (Table 1). In contrast to the results for Europe, neither the maximum HWMId nor the duration of the heat waves had a significant influence on the presence of stock price reactions in the U.S portfolios. Hence, *H2b* is rejected for the U.S. However, the two most recent heat waves in particular caused more noticeable stock price effects (Table 2). This may be explained by a higher sensitization to climate issues in recent years. As expected, U.S. firms with low environmental performance were the most heavily penalized by investors (Fig 3). Due to the reported comparative outperformance of high-scoring firms in Fig 3, *H3* is accepted for the U.S. sample.

Upon examining the aggregated results, more statistically significant stock price reactions occurred for the U.S. than for the European portfolios (Fig 3). This finding suggests a greater salience of heat waves in the U.S. stock market compared to Europe. Moreover, the most statistically significant stock price reactions take place within the shortest EW of [−1, +1], centered around the peak day. This finding highlights the important role of the peak day of heat waves as this day typically attracts the most public attention. Furthermore, over the whole sample, there are fewer stock price reactions for firms with high environmental performance, while firms with low environmental performance experienced the most stock price reactions. Overall, most stock prices were negatively affected across the entire sample (Fig 3).

## Discussion

### Synthesis and contributions

This study contributes to research methodology by presenting a pioneering fusion of methodologies from meteorology and finance. This innovative approach breaks new ground by integrating primary meteorological data with event study methodology in the context of efficient stock markets [70]. This methodological synergy not only enhances the robustness of financial decision-making in the face of climate change but also sets a precedent for interdisciplinary research. By doing so, it opens up novel pathways to understanding the intricate interplay between global climate phenomena and investor behavior. Thus, this study addresses the research gap highlighted by Venturini [22], emphasizing interdisciplinary collaboration with climate scientists to enhance the evaluation of climate risk threats to financial markets.

Moreover, this study contributes to climate science by meteorologically identifying the five most extreme European and U.S. heat waves, respectively, since 1979. The results derived from the applied HWMId method are supported by previous studies. For example, the European heat waves in 2010 and 2014 (Fig 2a) were also identified as among the strongest European heat waves in previous research. In particular, the 2010 heat wave was confirmed as the strongest on record over the European continent [57], and the European heat waves in 2018 and 2021 were also identified as major heat events by Lhotka and Kysely [71]. Despite its short duration (Table 2), the 2021 heat wave over northwestern parts of the U.S. is well-documented and has received significant attention [72, 73]. The longer durations of heat events observed in Europe compared to the U.S. are theoretically confirmed and explained by differences in atmospheric circulation patterns [74]. The alignment with previous research supports the application of the study’s grid-point method for continental analysis. Moreover, the result of an increasing frequency of heat waves as an imprint of global warming aligns with findings by the IPCC [6]. Through the meteorological analysis, this study addresses the research gap regarding the comparison of historical European and U.S. heat waves.

This study offers valuable contributions to the climate finance literature and practical insights for investors, firm managers, and policymakers, illustrating how heightened physical climate risk can diminish firm values. The authors posed the research question: *How do stock markets react to major European and U*.*S*. *heat waves*, *taking into account corporate environmental performance*? The event study results show that the five major heat waves in each region over the past 45 years have impacted stock markets, underscoring the urgent need for investors and firm managers to consider physical climate risks in their financial strategies. The negative reactions may stem from behavioral overreactions to salient heat events [40] or from the prior underpricing of heat risk, leading to market corrections [21]. For policymakers, these findings emphasize that investors are aware of physical climate risks, as demonstrated by the statistically significant stock market responses to each heat event. These negative market reactions highlight the urgent need for policies aimed at mitigating these risks and addressing their broader economic impacts. Moreover, the authors questioned whether the observed increase in the frequency of heat waves over Europe and the U.S. within the past four decades has led to increased awareness of global warming in the stock market. In the U.S., the stock market has been more responsive to recent heat waves, whereas in Europe, stock price reactions are influenced by their intensity and duration. Investors’ reactions to European heat waves possibly do not appear to have increased for more recent events because this region has already been a pioneer in environmental awareness for several years [75]. In contrast, the U.S. stock market’s growing attention to climate risks in recent years aligns with Schuster et al. [37] and their finding of a catch-up effect in the U.S. market compared to Europe. This aligned catch-up effect in climate risk recognition is further demonstrated by a generally higher frequency of statistically significant stock price reactions in the U.S. market. The assumption underlying the catch-up effect in climate risk recognition and pricing stems from historically higher levels of climate skepticism in the U.S. compared to Europe, followed by a gradual increase in sensitization [37]. Consequently, this pattern may also extend to the valuation of green and brown stocks in response to heat events. U.S. portfolios associated with high environmental performance exhibited a reduced likelihood of facing value losses, thus suggesting a potential hedging opportunity for investors and firm managers. This result aligns with the findings by Li [76], implying that firms with higher environmental, social, and governance (ESG) ratings are better equipped to adapt to increased physical climate risk exposures. Furthermore, the U.S. stock market shows a normative character by punishing firms with low environmental scores, resulting in value losses for contributors to climate change. This market behavior provides a financial foundation for U.S. climate policies by demonstrating the stock market’s capacity to support a green transition. Moreover, the study’s results align with those of Choi et al. [41] that investors sell brown stocks during heat periods. The stock price reactions to U.S. heat waves also confirm the finding by Huynh and Xia [40] of less market penalization for U.S. firms with superior environmental performance in times of salient physical climate risks (Fig 3). The outperformance of high-scoring firms in periods of high salience to physical climate risks in the U.S. demonstrates alignment among different proxies for environmental performance: this study utilizes the environmental performance ratings provided by LSEG, whereas Huynh and Xia [40] employ metrics by MSCI. Nevertheless, it is important to note the recent criticism directed at ESG rating providers for their lack of consistency, as highlighted by Berg et al. [77]. Contrary to expectations, the results for the European sample show the most positive stock price reactions for firms with low environmental performance and the most negative for firms with high environmental performance. Accordingly, for European stocks, investors in brown stocks (firms with low environmental performance) demand higher returns as compensation for holding riskier assets. In contrast, green stocks (firms with high environmental performance) deliver lower returns compared to the market, reflecting investors’ acceptance of reduced returns for non-pecuniary reasons. These explanations are in line with Pástor et al. [49]. Chiappini et al. [78] also previously identified a positive stock price reaction among sustainable firms in response to exogenous market shocks in the U.S., in contrast to a negative effect in Europe. Furthermore, Kaiser [43] suggests that sustainability performance has not yet been fully reflected in the market value of U.S. firms compared to European firms. Consequently, in Europe, corporate environmental performance grades may already be priced into stock returns and are therefore not perceived as a ‘new benefit’. In contrast, the U.S. market may have shifted its focus to the previously underemphasized environmental performance levels of firms, recognizing higher performance as advantageous during heat waves.

### Limitations and future research agenda

The novel insights from this study highlight four limitations and corresponding major avenues for future research.

First, the findings of this study suggest that, unlike in the U.S. sample, environmental performance is not a suitable proxy for hedging European stocks against physical climate risks, specifically heat risk. Future studies should further examine investor expectations to understand why high environmental performance is not enough to hedge European firm values against heat waves. The dilution effect detected in European environmentally high-scoring portfolios should encourage future research to find a suitable proxy that firm managers and investors can rely on. A more rigorous measurement of corporate environmental responsibility is required to protect firm values in light of the advancing environmental regulations in Europe [79].

Second, this study relies solely on environmental performance grades by LSEG as single proxy for corporate environmental responsibility. By investigating and comparing different proxies for environmental performance as potential hedges against salient physical climate risks, future research could contribute to the discussion on the divergence or convergence of ESG ratings [77, 80].

Third, this study compares portfolios based on corporate environmental responsibility. While this provides insights into investor preferences concerning individual firm performance, conclusions regarding sector sensitivities cannot be derived. The authors recommend that future research investigate the effects of these particular heat waves on various industry portfolios, specifically considering industries with high exposure to heat risk [29, 81].

Fourth, this study exclusively focuses on two highly developed, industrialized regions with significant market capitalizations: the U.S. and Europe. The authors recommend that future studies adopting this study’s interdisciplinary method encompass a broader range of geographical regions to gain further meteorological and stock market insights, particularly in developing countries. Researchers may wish to investigate the Global South, particularly regions that are especially vulnerable to physical climate risks [82].

## Conclusion

This study examined how the rising frequency of heat waves since 1979 has heightened stock market awareness of global warming. In Europe, stock price reactions are influenced by the intensity and duration of heat waves, whereas in the U.S., the market has shown greater responsiveness to recent heat waves, with superior corporate environmental performance mitigating heat risk. The study draws investors’, firm managers’, and policymakers’ attention to the fact that heat waves can significantly erode firm value.

## Supporting information

S1 Fig Major European heat waves from 1979 to 2023. This figure shows the HWMId for each year in the period 1979 to 2023 and for each grid-point on the land area within the European domain. The maps were created using the PlateCarree projection from the Cartopy Python package. Additional details for creating the figure can be found in the study’s minimal data set (see S1 File).
(TIF)

S2 Fig Major U.S. heat waves from 1979 to 2023. This figure shows the HWMId for each year in the period 1979 to 2023 and for each grid-point on the land area within the U.S. domain. The maps were created using the PlateCarree projection from the Cartopy Python package. Additional details for creating the figure can be found in the study’s minimal data set (see S1 File).
(TIF)

S1 File Minimal data set for the study ’Physical climate risk: Stock price reactions to the historically most extreme European and United States at waves since 1979’. The file contains supporting information regarding data accessibility and processing.
(DOCX)
