# Supplementary figures and images for: Physical climate risk: Stock price reactions to the historically most extreme European and United States heat waves since 1979
Source: PLoS One. 2025 Jan 24;20(1):e0318166. doi: 10.1371/journal.pone.0318166 (PMC11760027; doi:10.1371/journal.pone.0318166)

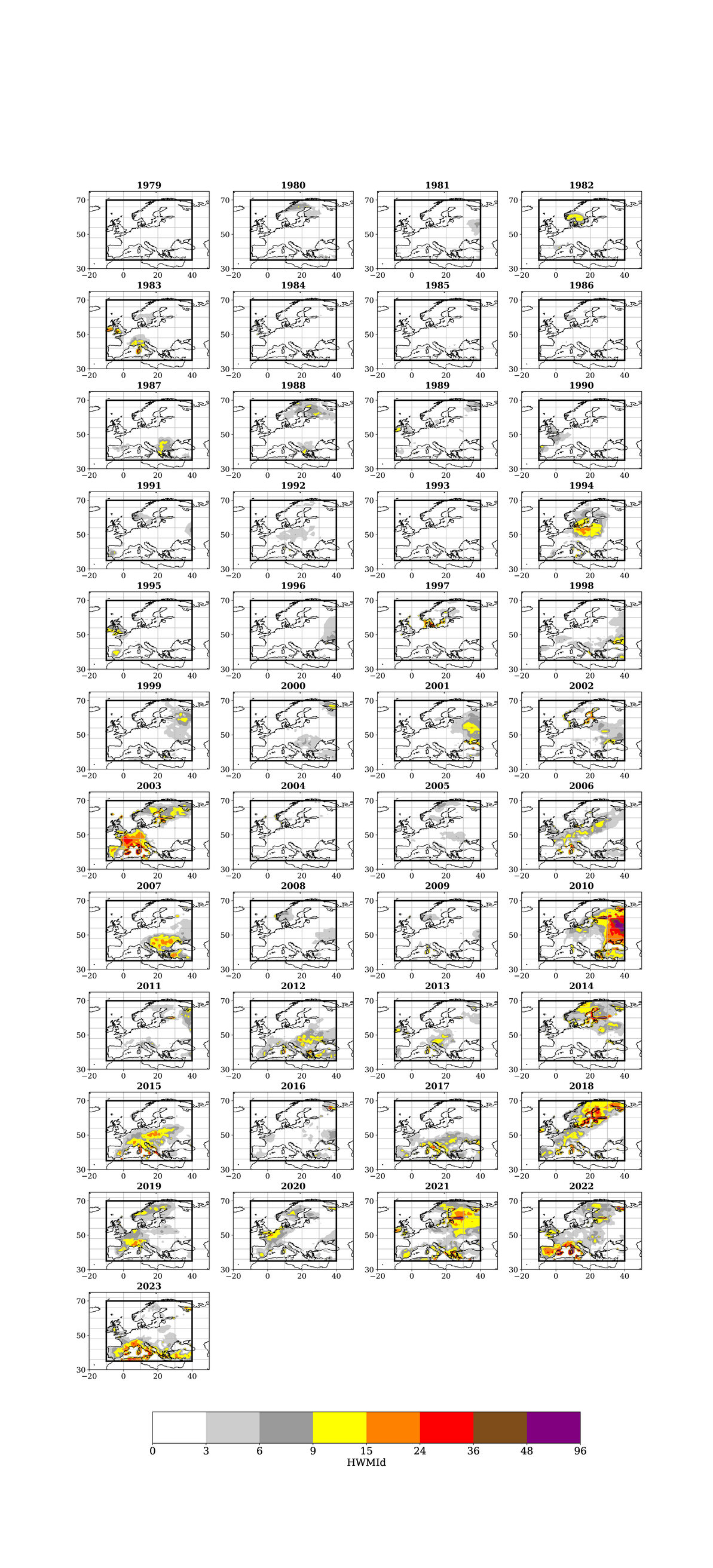

Supplement: S1 Fig — This figure shows the HWMId for each year in the period 1979 to 2023 and for each grid-point on the land area within the European domain. The maps were created using the PlateCarree projection from the Cartopy Python package. Additional details for creating the figure can be found in the study’s minimal data set (see S1 File). (TIF) [file pone.0318166.s001.tif]

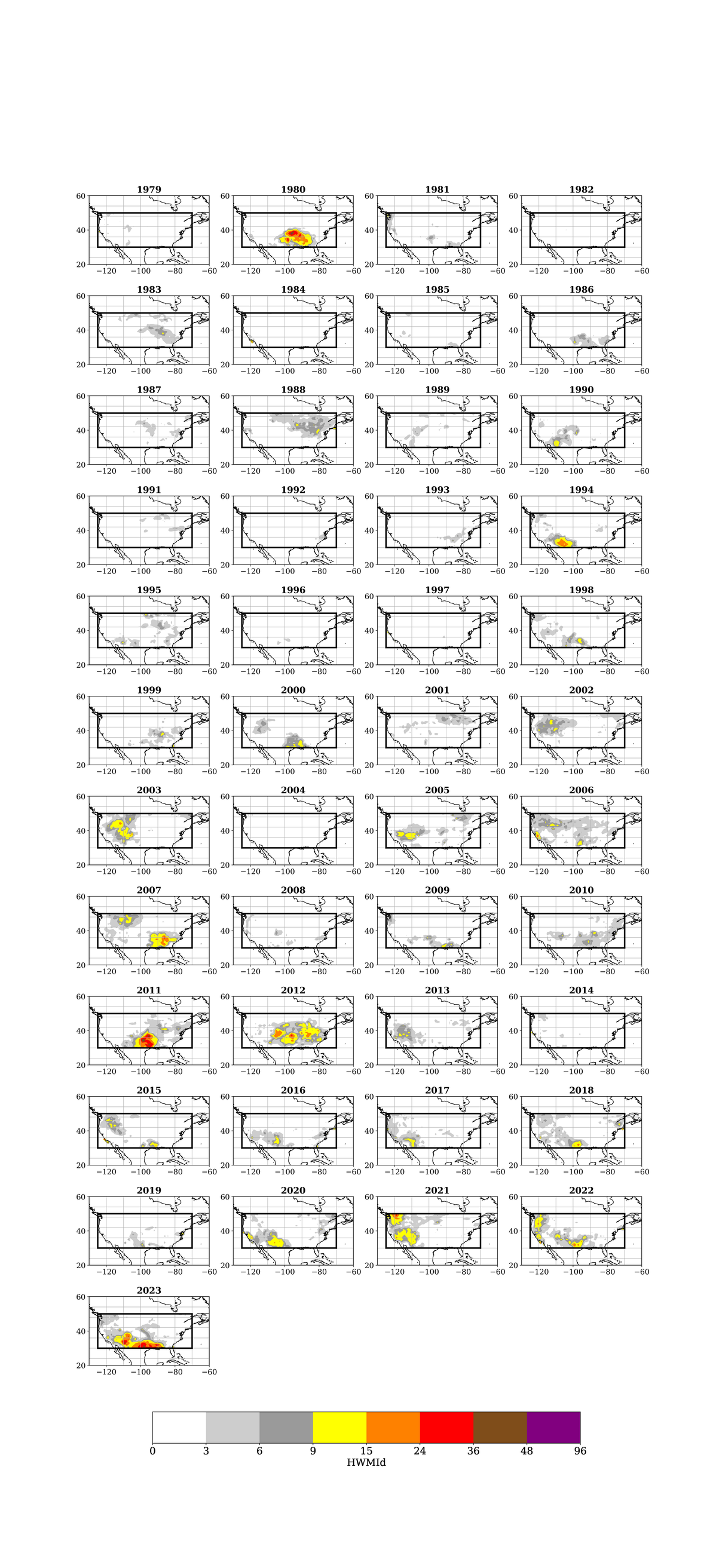

Supplement: S2 Fig — This figure shows the HWMId for each year in the period 1979 to 2023 and for each grid-point on the land area within the U.S. domain. The maps were created using the PlateCarree projection from the Cartopy Python package. Additional details for creating the figure can be found in the study’s minimal data set (see S1 File). (TIF) [file pone.0318166.s002.tif]
