# Supplementary material for: Physical climate risk: Stock price reactions to the historically most extreme European and United States heat waves since 1979
Source: PLoS One. 2025 Jan 24;20(1):e0318166. doi: 10.1371/journal.pone.0318166 (PMC11760027; doi:10.1371/journal.pone.0318166)
Supplement: S1 File — The file contains supporting information regarding data accessibility and processing. (DOCX) [file pone.0318166.s003.docx]

**Minimal data set for the study 'Physical climate risk: Stock price reactions to the historically most extreme European and United States heat waves since 1979'**

- The ERA5 reanalysis dataset, used in the HWMId analysis, is publicly accessible
  and can be downloaded from the European Centre for Medium-Range Weather Forecasts and the Climate Data Store (CDS):
  - <https://www.ecmwf.int/en/forecasts/dataset/ecmwf-reanalysis-v5>
  - <https://cds.climate.copernicus.eu/datasets/reanalysis-era5-single-levels?tab=overview>
- The Python code for calculating the Heat Wave Magnitude Index daily (HWMId)
  was obtained from the following GitHub repository: <https://github.com/julian28295/HWMID_analysis>
- Figures 1, S1, and S2 were created using open-source and freely available
  Python libraries/packages (<https://www.python.org/>), including:
- xarray: A package for handling large and multi-dimensional datasets (<https://docs.xarray.dev/en/latest/index.html>).
- matplotlib: A package for plotting and visualizing datasets (<https://matplotlib.org/>).
- cartopy: A package for visualizing datasets on global maps (<https://pypi.org/project/Cartopy/>). The PlateCarree projection was selected to generate the maps (<https://scitools.org.uk/cartopy/docs/v0.15/crs/projections.html>).

All the aforementioned references are sufficient to replicate Figures 1, S1, and S2.

- Data processing for the event study was conducted using the software tool Stata (<https://www.stata.com/>). The authors utilized Stata codes provided by Ullah et al. (2021) (please see Appendix A: <https://doi.org/10.1016/j.indmarman.2021.02.004>).
